# Cardiovascular health care and health literacy among immigrants in Europe: a review of challenges and opportunities during the COVID-19 pandemic

**DOI:** 10.1007/s10389-020-01405-w

**Published:** 2020-10-27

**Authors:** Bernhard Wernly, Sarah Wernly, Anthony Magnano, Elizabeth Paul

**Affiliations:** 1 Department of Internal Medicine II, Paracelsus Medical University Salzburg, Salzburg, Austria; 2 Department of Internal Medicine, General Hospital Oberndorf, Teaching Hospital of the Paracelsus Medical University Salzburg, Oberndorf, Austria; 3 St. Vincent’s Medical Center, Jacksonville, FL USA; 4 University of North Florida, Jacksonville, FL USA

**Keywords:** Migrants, Cardiovascular, Covid-19, SARS-CoV-2, Health literacy

## Abstract

**Objectives:**

Europe is a destination for many migrants, a group whose proportion of the overall population will increase over the next decades. The cardiovascular (CV) risk distribution and outcomes, as well as health literacy, are likely to differ from the host population. Challenges related to migrant health status, cardiovascular risk distribution and health literacy are compounded by the ongoing *coronavirus disease 2019* (COVID-2019) crisis.

**Methods:**

We performed a narrative review of available evidence on migrant CV and health literacy in Europe.

**Results:**

Health literacy is lower in migrants but can be improved through targeted interventions. In some subgroups of migrants, rates of cardiovascular disease (CVD) risk factors, most importantly hypertension and diabetes, are higher. On the other hand, there is strong evidence for a so-called healthy migrant effect, describing lower rates of CV risk distribution and mortality in a different subset of migrants. During the COVID-19 pandemic, CV risk factors, as well as health literacy, are key elements in optimally managing public health responses in the ongoing pandemic.

**Conclusions:**

Migrants are both an opportunity and a challenge for public health in Europe. Research aimed at better understanding the healthy migrant effect is necessary. Implementing the beneficial behaviors of migrants could improve outcomes in the whole population. Specific interventions to screen for risk factors, manage chronic disease and increase health literacy could improve health care for migrants. This pandemic is a challenge for the whole population, but active inclusion of immigrants in established health care systems could help improve the long-term health outcomes of migrants in Europe.

## Introduction

Non-communicable diseases (NCDs) are the primary cause (around 70%) of death worldwide. Among NCDs, cardiovascular disease (CVD) is the top cause of death, being responsible for about 50% of mortality (Cosentino et al. 2020; Knuuti et al. 2020).

The management and outcomes of NCDs have improved over the past few decades. The scientific community contributed to a better understanding and new pharmacologic and interventional treatment options (Cosentino et al. 2020; Knuuti et al. 2020). However, from both an individual level and public health perspective, disease prevention is preferable to managing established chronic disease. Health literacy is fundamental to successful prevention (Magnani et al. 2018).

Large-scale immigration of non-European refugees peaked in 2015 in Europe but is likely to continue in upcoming years. Immigrants differ from the host population with regard to genetics, baseline risk distribution, lifestyle and health literacy (Cainzos-Achirica et al. 2019). These differences affect the prevalence and incidence of NCDs. Also, access to health care is challenging for immigrants in some countries.

The United Nations defines a long-term migrant as a “person who moves to a country other than that of his or her usual residence for a period of at least a year.” Europe was a source of migration in the nineteenth century, but is now a destination for migrants. Between 2005 and 2010, the number of migrants living in Europe increased from 65 million to 70 million and is expected to continue to rise (Rechel et al. 2013). These 70 million migrants suffer from high rates of unemployment and low socioeconomic status and are, therefore, not only a humanitarian but also an economic and public health challenge (Rechel et al. 2013). Health care data on migrants in Europe is scarce for several reasons, including non-standardized definitions of migrants in health care systems and limited resources for public health research (Rafnsson and Bhopal 2008). However, the description of challenges and opportunities migrants pose for public health might be crucial for improving health care for all.

In December 2019, the new coronavirus *severe acute respiratory syndrome coronavirus 2* (SARS-CoV-2) emerged, causing *coronavirus disease 2019* (COVID-2019). Clinically, COVID-19 manifests similarly to influenza (symptoms such as fever, cough dyspnea, myalgia), but appears to have a higher risk of mortality than influenza, particularly in the elderly (Huang et al. 2020). The World Health Organization (WHO) declared the ongoing crisis a pandemic. As a consequence, policymakers reacted to COVID-19 with extensive public health measures, most notably non-pharmacologic intervention and physical distancing, which have been shown effective in prior pandemics (Markel et al. 2007). There is increasing evidence supporting the concept that patients with pre-existing CV conditions are at higher risk of death due to COVID-19 (Huang et al. 2020). Although COVID-19 primarily manifests as a respiratory disease, patients with COVID-19 die from inflammatory, CV and respiratory causes (Huang et al. 2020).

Both the disease itself and the social, public and economic consequences of the COVID-19 pandemic are likely to affect the lives of migrants throughout Europe. The integration of migrants in the European public and health care systems is likely to be affected by COVID-19. Furthermore, the successful management of the SARS-CoV-2 pandemic using non-pharmaceutical interventions depends on the compliance and participation of migrants.

We, therefore, aimed to summarize the evidence on CVD and health literacy of immigrants in Europe. Further, we evaluated the relation between the ongoing COVID-19 pandemic and migrant CV risk distribution and health literacy.

## Methods

We performed a narrative review of CV risk distribution and health literacy in migrants in the European Union, including Switzerland, Norway and the United Kingdom. Migrants, immigrants and refugees included people from all developing countries as well as Eastern European countries and the Balkan region, which are not part of the European Union. This group was analyzed in light of the challenges related to the ongoing COVID-19 pandemic. We performed a search on Medline and Google Scholar using the keywords “migrant,” “refugee,” “cardiovascular,” “diabetes,” “hypertension,” “non-communicable disease,” “SARS-CoV-2,” “COVID-19” and “COVID” in different combinations. In total, the search yielded 2183 manuscripts. After deletion of duplicates and reports not concerning countries of the European Union, as well as manuscripts considered to be outdated based on our subjective clinical/public health expertise, 185 references were screened in depth. Also, reviews and statements on the topics of health literacy and migrant health were screened for relevant literature. Further, we screened co-citations using the CoCites algorithm for other relevant manuscripts. Based on available evidence and our judgment, the final 59 manuscripts were included in this narrative review.

## Results

### Cardiovascular disease in migrants

CVD is highly prevalent and leads to high mortality, morbidity and costs in both developing and developed societies (Benjamin et al. 2019). However, the rates of CVD also differ within Western and non-Western countries, as the distribution of risk factors and preventive measures of local health care systems vary (Agyemang et al. 2009; Rechel et al. 2013). Moreover, the economic capabilities of health care systems to provide CVD management according to the latest guidelines differ and likely contribute to differential outcomes worldwide (Cosentino et al. 2020; Knuuti et al. 2020).

The European host societies are more often urban and evidence higher levels of environmental pollution (Sohail et al. 2015). The host societies also promote behavioral changes among refugees, such as a sedentary lifestyle, which consequently changes the CV risk profile of migrants (Sohail et al. 2015). Depending upon the host country and the degree of integration, access to health care and the living conditions of migrants can influence outcomes in either first-generation or later-generation migrants or in none of the migrants (Raymundo et al. 2020).

Migrant populations are difficult to characterize, since their situations vary greatly. In one review, the rates of ischemic heart disease and stroke were heterogeneous among migrants in Europe (Sohail et al. 2015). Migrants from the Middle East and South Asia exhibited similar or higher rates of CVD and stroke compared to the host population (Sohail et al. 2015), in contrast to migrants from Northern Africa, who may have a lower rate of stroke. In another study from the Netherlands, South American migrants had higher stroke rates, while North African migrants demonstrated lower risk for CVD (Bos et al. 2004). This finding of lower risk for CVD in North African migrants was confirmed by a Spanish study, which reported lower rates in North African populations as compared to higher rates in Asian and sub-Saharan African groups (Regidor et al. 2009). Of note, the living conditions in the host countries are an additional contributing factor for differential rates of CVD, as South Asian migrants were at an even higher CVD risk than their relatives in the country of origin (Bhatnagar et al. 1995). In the RODAM [Research on Obesity & Diabetes among African Migrants] study, sub-Saharan African migrants in Europe showed higher rates of CVD than their counterparts living in a rural environment (Agyemang et al. 2016). While some of the effects might also be attributable to rural versus urban environments, the differences between migrants and their peers in an urban home country setting were small (Agyemang et al. 2016). Finally, the CVD outcomes and mortality of migrants differ between European host countries (Bhopal et al. 2012; Sohail et al. 2015). Interactions between the country of origin, host country and rural versus urban environment all play a role in determining CVD risk (Agyemang et al. 2016).

In sub-Saharan African migrants, hypertension is consistently more prevalent than in host populations (Agyemang et al. 2013; Commodore-Mensah et al. 2014; Modesti et al. 2014). In the Netherlands, the rates of hypertension in male migrants of sub-Saharan African origin was above 60%, compared to 34% in Dutch males (Agyemang et al. 2015). Studies across Europe have confirmed the finding of a higher prevalence of hypertension in immigrants (Modesti et al. 2016). Also, the disease was less successfully managed in migrants compared to the host population, which could indicate worse access to health care but also compliance problems (Agyemang et al. 2005; Agyemang et al. 2015).

In addition to hypertension, rates of type 2 diabetes are higher in migrants (Meeks et al. 2016). Diabetes is a key risk factor for CVD, and its management is difficult and usually long-term in nature (Pernow et al. 2019). Strikingly, diabetes is more prevalent among virtually all ethnic migrant groups (Agyemang et al. 2009; Sohail et al. 2015). A meta-analysis by Meeks et al. recently confirmed this finding and found higher rates of diabetes among Asian, Middle Eastern and African migrants compared to European host populations (Meeks et al. 2016). Mainly migrants from South Asia suffer from very high rates (3–6 times) of diabetes compared to European host populations (Meeks et al. 2016). Moreover, the onset of diabetes is not only more common but occurs more than 10 years earlier in immigrants (Snijder et al. 2017). Migrants are more likely to suffer from both micro- and macrovascular complications related to diabetes (Vandenheede et al. 2012). In one study, diabetes mortality was almost twice as high in migrants as in European host patients (Vandenheede et al. 2012). Both the living conditions in the host countries and genetic factors may explain this finding (Galbete et al. 2018). The rates of obesity are higher in migrants as well (Meeks et al. 2016). Also, differential patterns in diets, physical exercise and socioeconomic “dys-stress” likely contribute to the higher burden of diabetes in immigrants (Cosentino et al. 2020). However, these factors might not be enough to explain the differences in prevalence and outcome, and a deeper understanding of both access to health care but also genetic and epigenetic factors is crucial for fully evaluating the observed differences. Given the complex and interdisciplinary management of diabetes, this finding underscores the potential impact on European health care systems (Cosentino et al. 2020).

This conundrum reflects the multifactorial pathogenesis of CVD, which is the common final pathway of arterial hypertension, obesity, diabetes, socioeconomic factors and behavioral factors—such as physical exercise and nutrition—as well as genetic factors (Cosentino et al. 2020; Knuuti et al. 2020). These are all likely to either differ between migrants and the host population or be influenced by the migration and the subsequent changes in living conditions, income, access to health care and environmental factors.

### The healthy migrant effect

Migrant health is not a unidimensional subject. There is also evidence for the so-called healthy migrant effect, describing lower rates of CV risk distribution and mortality in migrants as compared to the host population (Delgado-Angulo et al. 2020; Gkiouleka and Huijts 2020). In a Danish study including more than 50,000 migrants and 200,000 age- and sex-matched Danes, the mortality rates were consistently lower in immigrants (Norredam et al. 2012). A Swedish register study supports this finding of lower CV events in migrants (Helgesson et al. 2019). Byberg et al. observed a similar pattern in a large register evaluating CVD incidence and mortality in 114,331 migrants matched 1:6 to persons born in Denmark: migrants had lower rates of death, and the subgroup of family-reunified migrants had lower rates of CVD (Byberg et al. 2016).

Some studies have reported that the observed differences in CVD risk between migrants and the host population decline in a time-dependent manner (Harding et al. 2008). In the Netherlands, North African migrants lost the advantage of lower CVD over time as the gap closed (van Oeffelen et al. 2014). These findings of increasing CVD rates in migrants over time is supported by intergenerational studies (Sundquist and Li 2006; van Oeffelen et al. 2013). In Sweden, second-generation migrants tended to exhibit higher rates of CVD than first-generation migrants, and the risk almost converged to the rates of the host population (Sundquist and Li 2006).

### Health literacy in migrants

Health literacy is an essential cornerstone of health promotion (Kickbusch 1997). Nutbeam proposed three levels of health literacy (Nutbeam 2000). First, functional health literacy relates to basic knowledge, including literacy, arithmetic, an understanding of disease and health services. Second, interactive health literacy corresponds to the social skills necessary for communication with health care providers. Third, critical health literacy refers to a broader knowledge of health-relevant information and the ability for informed decision-making (Nutbeam 2000). The health literacy of migrants could be impaired—but also improved through tailored interventions—on all of these levels (Fernandez-Gutierrez et al. 2018). Of note, a systematic review found interventions to be effective in increasing health literacy in immigrants (Fernandez-Gutierrez et al. 2018). However, specific programs targeting health literacy in migrants are scarce and not well studied, and there is likely both opportunity and demand for improvement (Jones et al. 2011).

Health literacy is key to successfully preventing and managing NCDs (Magnani et al. 2018). Interestingly, some studies indicate high awareness of NCD risk factors among migrants (Agyemang et al. 2014). The rates of treatment were higher in some groups of migrants compared to the respective host population (Agyemang et al. 2014). Still, there are substantial data indicating suboptimal management and control of CVD risk factors in migrants (Agyemang et al. 2014). Therefore, language barriers, differential perception of risk factors and concepts of health and disease are likely to contribute to a lack of adequate health care in migrants (Nutbeam 2000).

Health care providers need to recognize these differences on an individual basis. From a broader perspective, policymakers need to consider addressing and reaching out to migrants in innovative ways (Jervelund 2018). For example, in the United States, blood pressure measurement in barbershops, a popular social gathering place among American black men, was shown to improve control of arterial hypertension in this population (Rader et al. 2013). Patients screened for diabetes in barbershops evidenced higher rates of impaired glucose tolerance compared to the general population (Osorio et al. 2020).

### Impact of migrant cardiovascular diseases and health literacy during the COVID-19 pandemic

The ongoing COVID-19 crisis will affect humans across the globe as the epidemiology of this virus continues to unfold. However, it becomes increasingly clear that CVD constitutes a significant risk factor for severe illness (Zheng et al. 2020). The specific pathways of this association are the subject of ongoing research. Still, protecting the vulnerable will be a key strategy in controlling COVID-19 (Verity et al. 2020). Therefore, identification of patients at high risk for severe life-threatening COVID-19 is necessary.

Migrants evidence higher rates of CVD, particularly hypertension and diabetes, which have been linked to adverse outcomes in COVID-19. Therefore, the protection of these individuals is important. However, extending such protection relies on educating these patients about the new disease. Patients need to understand the risks of infection, how to limit exposure and who is at risk. As a result of the described limitations in health literacy but also language barriers, protecting migrants at risk will be challenging (Spiegel et al. 2020). Specifically, approaches in different languages need to be tailored to enable the public health systems to reach out to these patients (Greenaway et al. 2020).

Migrants are more likely to live in areas with greater population density, both in migrant camps and in European urban areas (Costa and de Valk 2018). The basic public health measures, including physical distancing, hand hygiene, and quarantine of symptomatic individuals and contact persons, are difficult to implement under these conditions (Maroko et al. 2020). Therefore, the distribution of migrants from camps to other, less densely populated areas with access to adequate housing and hygiene is now not only a philanthropic, but also a public health demand (Hargreaves et al. 2020). These measures, mitigating the spread of the SARS-CoV-2, will improve the health not only of migrants but of all inhabitants (Brandenberger et al. 2020). As long as migrant camps persist, disease-specific management should be available: Testing, tracking and tracing of both migrants inside and workers from outside have to be in place to avoid the introduction and transmission of SARS-CoV-2 (Brandenberger et al. 2020). Information about the virus and non-pharmacologic intervention measures should be available in the migrants’ native language, partly as an important countermeasure against fake news spreading via social media (Islam et al. 2020). Increasing the health literacy in newly arrived migrants could increase the acceptance of non-pharmacologic intervention, help to detect and isolate infected individuals and protect the vulnerable (Christie and Ratzan 2020).

Migrants are over-represented in the European homeless population (Anh Ly et al. 2020). For homeless persons, hygiene measures, self-isolation if symptomatic and social distancing may be nearly impossible (Bhopal 2020; Tsai and Wilson 2020). Oftentimes, homeless migrants sleep in crowded institutions during the winter. In this setting, transmission is likely and mitigation complicated (Bhopal 2020; Tsai and Wilson 2020).

## Limitations

Limitations of this review include its narrative design. Further, the terminology for immigrants is not precise and the population is highly diverse—in demographics, home country and host country. Thus, analysis as a single group based on their commonality as immigrants is an inherent oversimplification. Moreover, the distribution of source and host countries evolves over time within the growing European Union. Therefore, the literature selection was based on both structured review of available evidence and our subjective judgment of the suitability of the manuscripts reviewed here.

## Conclusion

Migrants and the European host population differ regarding CVD risk distribution and outcomes. Both biological (genetic, epigenetic) and non-biological (socioeconomic, behavioral) factors contribute to the observed differences. CV migrant health and risk factors are a multidimensional matter. Some immigrants do have higher rates of some CVD, most importantly hypertension and diabetes. Lower rates of health literacy and signals towards worse control of chronic diseases in migrants could lead to significantly worse outcomes in this population. However, in other migrant subgroups, CV outcomes are even better than the host population. This “healthy migrant effect” is likely multifactorial but could include genetic reasons, the age-distribution, a selection bias (“migration of the fittest”) and lifestyle factors. This effect was predominantly described in the first generation of migrants, and there is evidence suggesting that the healthy migrant effect diminishes over time. The European population should try understanding and integrating the reasons behind better health in some migrants. Implementing these measures in the whole community could benefit public health.

Migrants are both an opportunity and a challenge for European public health. Young and healthy migrants will likely contribute workforce, gross domestic product and wealth necessary to provide resources for the public health. Tailored programs to increase health literacy, screen for risk factors and manage chronically ill migrants such as the American barbershop interventions could improve outcomes. While the European host population does have a strong tradition of gathering in barbershops, other similar environments popular with immigrants could be a stage for basic health care and health literacy interventions.

During the pandemic, the European Union may benefit from granting migrants universal access to the local health care systems. Excluding migrants from health care likely decreases testing and tracing in this population group, which could counteract local containment, mitigation and suppression strategies. Another concern during the COVID-19 pandemic is that patients with chronic illnesses may not seek care due to social distancing measures and restrictions in access to care. A delicate balance exists between ensuring social distancing to minimize SARS-CoV-2 infections and promoting necessary contact between patients and health care providers. The application of e-health and telemedicine might be limited in migrants because of socioeconomic factors, language barriers and health literacy (Hollander and Carr 2020). Thus, emerging methods of physically distanced care for the host population may be much more difficult in migrants. Once a vaccination is available for SARS-CoV-2, health literacy might be key to promoting vaccination among immigrants, although the role of health literacy and vaccination hesitancy and acceptance is a subject of concern (Lorini et al. 2018).

The higher rates of risk factors and the lower rates of health literacy are a significant concern for migrants. The younger age of migrants, especially those who arrived recently, could constitute an advantage for the aging European populations. Younger patients have lower rates of severe illness due to COVID-19 and may contribute to herd immunity, which may ultimately be necessary to protect the vulnerable. During the pandemic, the universal inclusion of migrants in health care systems could help to ensure testing, tracking and tracing in the whole population. European societies need to provide both care and information to immigrants. This pandemic is a challenge for the whole population, but the necessary active inclusion of immigrants in the health care systems could also help with the long-term integration of migrants in Europe.
